# Challenges and Best Practices in Modeling Anisotropic Stresses in Soft Polymorphic Materials

**DOI:** 10.1021/acsphyschemau.5c00141

**Published:** 2026-01-08

**Authors:** Jelto Neirynck, Sander Geerinckx, Sven M. J. Rogge

**Affiliations:** Center for Molecular Modeling (CMM), 26656 Ghent University, Technologiepark-Zwijnaarde 46, 9052 Zwijnaarde, Belgium

**Keywords:** Cauchystat, stress control, molecular dynamics, MIL-53, COF-5, phase transition, anisotropy

## Abstract

Soft polymorphic materials, such as metal–organic frameworks (MOFs) and covalent organic frameworks (COFs), often display distinct anisotropy. Yet, their phase transition behavior has been predominantly characterized under isotropic stimuli, such as temperature or pressure variations, up to now. In this work, we employed the Cauchystat to investigate how MIL-53­(Al) and COF-5, two prototypical soft porous crystals, respond to anisotropic stresses instead. For MIL-53­(Al), we showed that normal stresses induce a phase transition already at stresses below the critical hydrostatic pressure, depending on the directionality of the applied stress. For COF-5, we determined the critical shear stress needed to induce a layer instability, leading to delamination. In both cases, we highlighted the importance of selecting adequate values of the Cauchystat control parameters to obtain accurate predictions. Based on these insights, we formulated best practices to simulate phase transitions in soft porous crystals under nonhydrostatic loadings, which is required for, e.g., nanosensors and -dampers.

## Graphical abstract

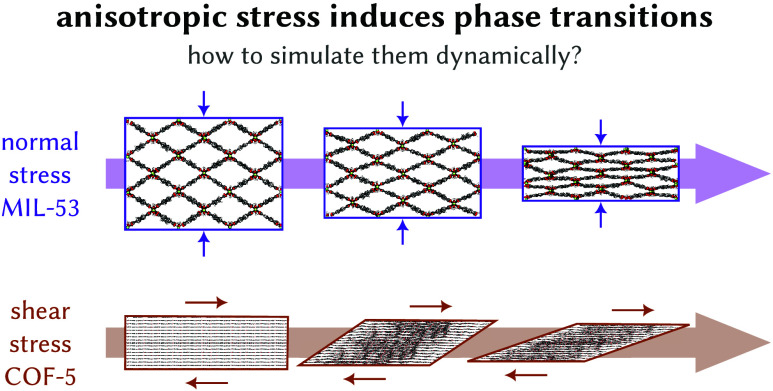

## 1 Introduction

Many solid-state materials change their structure *anisotropically*, even when stimulated under *isotropic* thermodynamic conditions such as temperature or pressure changes, due to the directionality of interactions between the material’s constituents and the diversity in interaction strengths.^1–3^ A noteworthy example is provided by MIL-53­(Al),^4^ the metal–organic framework (MOF) shown in Figure 1a. Subjecting this MOF’s large-pore (lp) phase to a pressure of 13–18 MPa induces a single-crystal-to-single-crystal phase transition to the closed-pore (cp) phase;^5^ a similar phase transition can be induced by temperature variations.^6^ During this lp-to-cp transition, all three cell vectors respond differently: the cell vector along the inorganic [Al­(μ_2_–OH)]_*n*_ chain remains virtually unchanged, whereas the one along the *z* and *x* axes contracts and expands, respectively, as illustrated in Figure 1a.^5^ While such a pronounced anisotropy—even leading to negative linear compressibility in this case^7^—is extremely rare in most materials, it is encountered more frequently in many porous framework materials, such as MOFs and their fully organic counterparts, covalent organic frameworks (COFs).^8^ Especially for these types of materials, the commonplace practice of investigating their response to isotropic stimuli alone can obscure the intriguing behavior they may exhibit under anisotropic stimuli. For instance, while ample research has focused on determining the critical pressure needed to induce an lp-to-cp transition in MIL-53­(Al) through applying a hydrostatic stress,^5,9^ it remains unclear to what extent this critical stress can be lowered by inducing this transition through normal stresses instead, as indicated by the purple arrows in Figure 1. In this manuscript, we aim to fill this gap by exploring how anisotropic stresses can induce phase transformations in two prototypical MOFs and COFs, even at stresses below the reported hydrostatic transition pressure.

**Figure 1:**
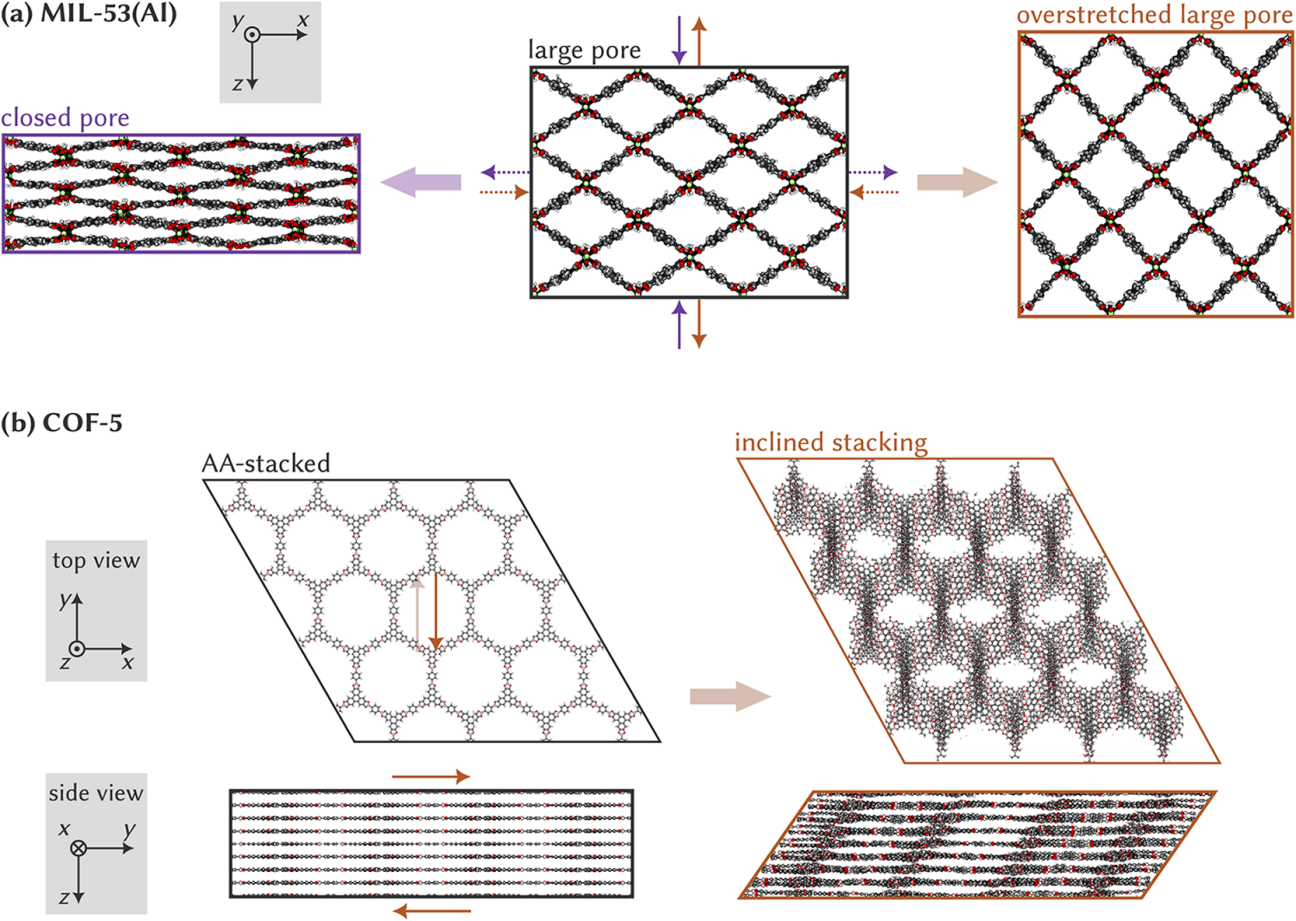
Atomic structures of (a) MIL-53­(Al) and (b) COF-5, the latter both in top and side view. For MIL-53­(Al), four normal stresses are indicated on the large pore cell: a compressive *z* stress (solid purple) or a tensile *x* stress (dotted purple) lead to a transition to the closed pore state, whereas a tensile *z* stress (solid brown) or a compressive *x* stress (dotted brown) lead to a transition to the overstretched large pore state. For COF-5, the brown shear stress steers the AA-stacked unit cell to one with inclined stacking. Color code: aluminum (lime), oxygen (red), carbon (gray), boron (pink), and hydrogen (white).

Computer simulations have proven pivotal to describe, rationalize, and design the counterintuitive behavior of MOFs and COFs, including the effect of defects on the amorphization pressure in the UiO-66 class of materials,^10,11^ negative gas adsorption in the DUT-49 family,^12,13^ the impact of crystal size on the phase transition mechanism,^14,15^ and the dynamic layer stacking in 2D COFs.^16–18^ In all these cases, the advantage of these *in silico* approaches lies in the fact that they can isolate how a given structural variation in a material, e.g., a metal or linker substitution, impacts the material’s stimuli-responsiveness, while keeping all other potentially confounding variables constant.^2^ In all examples mentioned above—and this is true for the broader MOF and COF field—however, the investigated thermodynamic stimuli were isotropic in nature, with structural responses induced by anisotropic stimuli being rationalized through thermodynamic models but not simulated directly. For instance, Neimark and co-workers introduced the concept of “critical adsorption stress” to explain when guest adsorption or desorption leads to phase transitions in MIL-53­(Al), among other materials.^19^ Key to this idea is that guest molecules adsorbed in a MOF’s pores exert an anisotropic stress on the pore wall, which at a certain point overcomes the critical threshold the pore can withstand.^19^ While in this case the transition-inducing stimulus—the adsorption stress—is anisotropic, the stimulus controlled during the simulation—the gas pressure—remains isotropic in nature. Another approach consists of controlling the anisotropic strain instead of the stress,^20^ or in applying anisotropic forces on well-defined atoms in a nonperiodic simulation.^21^ While in these cases anisotropic stimuli are adopted, the controlled stimulus is not the stress. Finally, an alternative thermodynamic approach is commonly adopted in 2D COFs, nanoporous materials consisting of covalently bound 2D layers that interact with one another through weak dispersion and electrostatic interactions.^22,23^ Due to these weak interlayer interactions, adjacent layers in 2D COFs can stack dynamically, resulting in different layer configurations, such as the AA-stacking and inclined stacking shown in Figure 1b for COF-5.^16,18,24–26^ In recent years, several groups derived free energy surfaces to characterize this stacking behavior, providing insight into the relative stability of different layer stackings and the barriers separating them.^16–18,26^ While these barriers inform us about the free energy required to induce a transition from one stacking configuration to another, they leave unanswered the fundamental question of which shear stress is needed to cause such a transition. Herein, for the first time, we answer these fundamental questions by directly applying normal stresses in MIL-53­(Al) and shear stresses in COF-5, thereby revealing how these critical stresses depend on the direction of application. In this way, we allow for a direct link between these constant-stress simulations and the constant-stress experiments to which these materials could be subjected.

A variety of algorithms exist to control the pressure during molecular dynamics (MD) simulations, including the Andersen,^27^ Berendsen,^28^ Hoover,^29–31^ Langevin,^32,33^ Martyna-Tuckerman-Tobias-Klein,^34,35^ and Bussi-Zykova-Parrinello barostats.^36^ In many cases, these algorithms allow for anisotropic material responses—i.e., both the simulation cell volume and its shape can vary—but are limited to controlling only the isotropic pressure as the input variable. These pressure control algorithms are widely available in most MD engines, including LAMMPS,^37^ CP2K,^38^ VASP,^39–41^ i-PI,^42^ and DL_POLY.^43^ In contrast, anisotropic stress algorithms that control the full stress tensor are scarce, contributing to the limited number of anisotropic stress studies. Noteworthy examples of stress control algorithms include the Parrinello–Rahman barostat^44^ and the much more recent Raiteri-Gale-Bussi barostat,^45^ applicable only in the elastic regime around a predefined reference cell. While the Parrinello–Rahman barostat is the method of choice for stress control in many MD engines, it does not control the true stress or Cauchy stress, as demonstrated in ref 46 and discussed in more depth in Section 2. This distinction is crucial in cases where the applied anisotropic stress induces large structural deformations beyond the elastic regime, such as the aforementioned phase transitions in MOFs and COFs;^46^ in these cases, also the Raiteri-Gale-Bussi barostat is no longer applicable. For this reason, Miller et al. developed an adaptive algorithm that controls the correct Cauchy stress.^46,47^ This Cauchystat, which has since been implemented in LAMMPS,^37^ has subsequently been employed to study martensitic phase transitions of a nickel–aluminum alloy,^46^ stress-induced yielding in a Lennard-Jones mixture,^48^ copper thin film growth,^49^ and thermal expansion in copper.^50^ While yielding promising results, these previously studied materials are harder and denser than MOFs and COFs, making them less sensitive to potential deficiencies in the stress algorithm. Earlier, we used soft porous crystals instead to demonstrate shortcomings in isotropic barostats that would remain obscured in harder materials, including the propensity to induce phase transitions at pressures substantially below the correct transition pressure.^9^

In this study, we therefore critically investigate the applicability of the Cauchystat to induce stress-induced phase transitions in two challenging classes of soft materials. In MIL-53­(Al), we determine the normal stress needed to induce transitions to either the closed-pore or overstretched large-pore phase (Figure 1a), while in COF-5, the critical shear stress to induce layer instability (Figure 1b) is investigated. In both cases, we relate the fluctuations in the instantaneous Cauchy stress, controlled by the Cauchystat, to the observed changes in cell parameters before, during, and after the induced phase transformations. In addition, we thoroughly discuss the sensitivity of the obtained results on the Cauchystat parameters and the stochasticity of the observed phase transitions. Based on these observations, we identify best practices when using the Cauchystat for these classes of soft anisotropic materials.

## 2 Methods

### 2.1 Cauchystat

Stress is a second-rank tensor that is defined as a force acting on a material’s surface, divided by the area of that surface. However, this is not a unique definition when a material deforms under said stress.^46,47^ Consider a subbody of a material, as sketched on the left in Figure 2. In the context of MD simulations, this refers to the simulation cell at a specific time *t*_0_, defined by the cell matrix **h**_0_, which contains the three periodic cell vectors at that time instant. When subjected to a certain stress, this reference cell **h**_0_ = **h**(*t*_0_) responds by deforming to the cell matrix **h**(*t*) at a later time *t*. Following Cauchy and Born, this time-dependent deformation can be described by the deformation gradient **F**(*t*) = **h**(*t*) **h**_0_^–1^, a second-rank tensor.^51,52^

**Figure 2:**
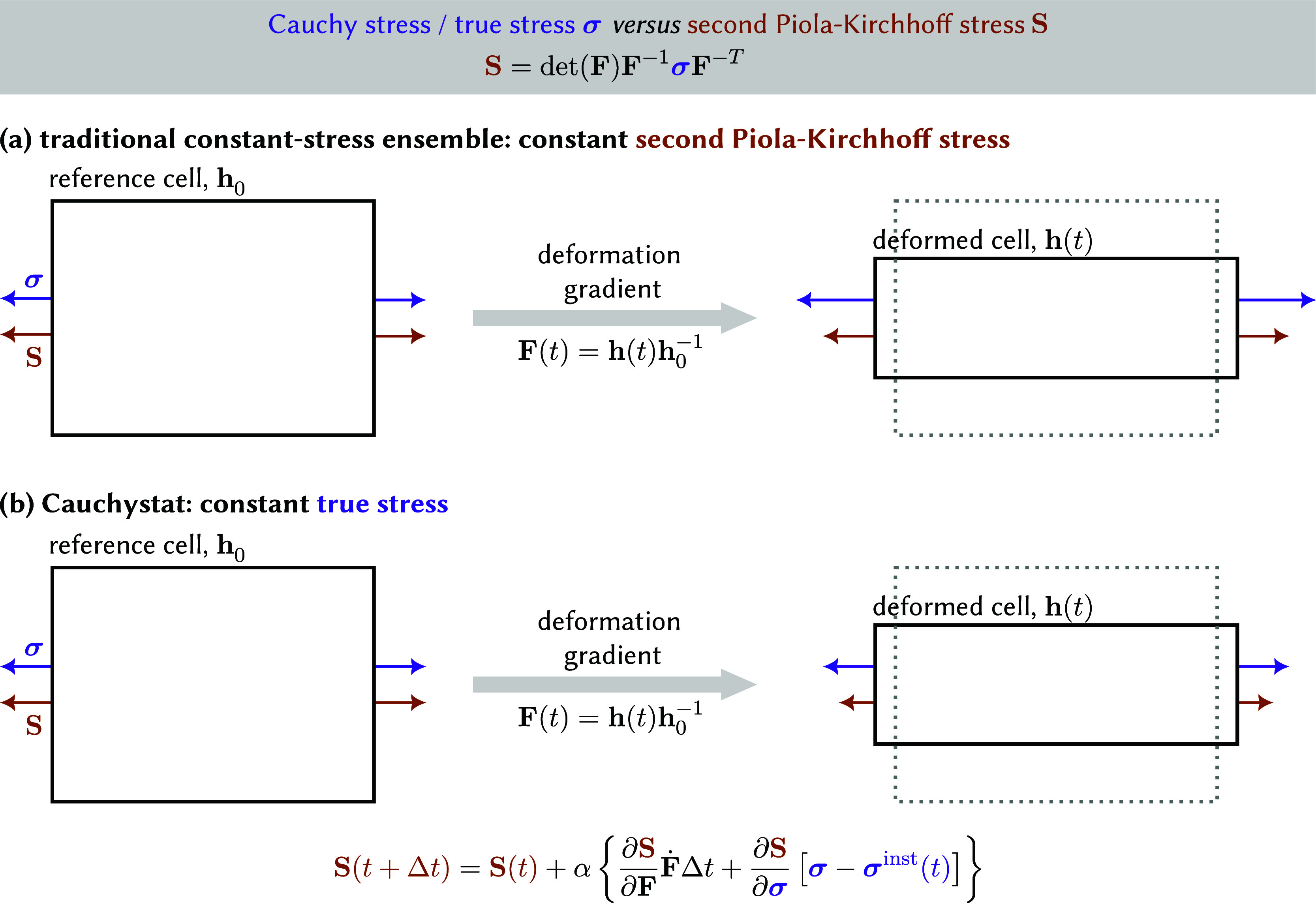
Comparison of stress tensors during a “constant–stress” MD simulation. (a) In traditional stress control, the second Piola-Kirchhoff stress, which is related to the engineering stress, is kept constant. This results in a varying true stress when the simulation cell deforms. (b) The Cauchystat aims to simulate the system under a constant true stress while controlling the second Piola-Kirchhoff stress, which therefore needs to be updated throughout the simulation.

The various definitions of stress differ in how the surface area is defined on which the force acts. If one uses the deformed area and acknowledges that the material deforms in the process, the true stress, also known as the Cauchy stress **σ**, is obtained. However, one often uses the undeformed reference area instead since it is available *prior* to the actual stress experiment, whereas the deformed area can only be obtained during the experiment or simulation. Defining stress based on the reference area leads to the definition of the engineering stress, also known as the first Piola-Kirchhoff stress **P**, which is related to the second Piola-Kirchhoff stress **S** = **F** ^–1^ **P** by mapping the forces on the deformed cell back to the forces on the reference cell. These stress metrics are related to one another through

1 $$S=\det\left(F\right)F^{-1}\sigma F^{-T}$$

with **F** ^–1^ and **F** ^*T*^ the inverse and transpose of the deformation gradient **F**, and **F** ^–*T*^ = (**F** ^–1^)^*T*^. Only in the undeformed case is **F** = **1**, and do these stress tensors coincide.

Figure 2a depicts schematically how these different stresses evolve during an MD simulation when the stress is applied through the often-used Parrinello–Rahman barostat. For the reference cell, at the onset of the simulation, the second Piola-Kirchhoff stress **S** (brown arrows) and the Cauchy stress **σ** (purple arrows) coincide. As demonstrated by Miller et al., the equations of motion derived through the Parrinello–Rahman barostat control the second Piola-Kirchhoff stress **S**.^46^ Hence, when the material deforms during the “constant-stress” MD simulation, and **F**(*t*) ≠ **1** as a result, keeping the applied second Piola-Kirchhoff stress **S** constant will necessarily result in a varying true stress **σ**. This is undesired in case one is interested in probing a material’s behavior under a constant true stress, i.e., in the targeted (*N*, **σ**, *T*) ensemble.

To control the Cauchy stress during an MD simulation, instead, the Cauchystat builds on the Parrinello–Rahman barostat by updating the applied second Piola-Kirchhoff stress after each time step Δ*t* in the simulation, according to the equation

$$S\left(t+\Delta t\right)=S\left(t\right)+\alpha \left\{\frac{\partial S}{\partial F}Ḟ\left(t\right)\Delta t+\frac{\partial S}{\partial \sigma }\left[\sigma -\sigma^{\mathrm{inst}}\left(t\right)\right]\right\}$$ 2

In this equation, **Ḟ**(*t*) is the time derivative of the deformation gradient and **σ** ^inst^(*t*) is the instantaneous Cauchy stress at time instant *t*, while α is a proportional gain parameter with a recommended value between 0.01 and 0.001 in LAMMPS. In the limit α → 0, the original Parrinello–Rahman barostat with the thermodynamically correct stress fluctuations is obtained, but the Cauchy stress is no longer controlled.^46^ In contrast, greater values of α allow for larger corrections to the applied second Piola-Kirchhoff stress due to either significant changes in the deformation gradient or significant deviations between the desired Cauchy stress **σ** and its instantaneous value **σ** ^inst^(*t*). This leads to the situation depicted in Figure 2b, where the second Piola-Kirchhoff stress **S** varies throughout the Cauchystat-controlled MD simulation to maintain a constant target Cauchy stress **σ**. One of our main goals in this manuscript is identifying whether there exists an optimal value of α when considering stress-induced phase transitions in soft polymorphic materials, such as MOFs and COFs.

### 2.2 Computational Details

Throughout this manuscript, a 3 × 6 × 3 supercell of MIL-53­(Al) containing 4104 atoms has been employed, with the second dimension lying along the inorganic chain. As demonstrated in Section S1 of the Supporting Information, a cell of this size is needed to reproduce the lp-to-cp transition pressure under a hydrostatic pressure.^5,9^ To allow comparison with literature, the MIL-53­(Al) cell parameters are mapped back to a 1 × 2 × 1 cell. For COF-5, a 4 × 4 × 4 supercell containing 12,288 atoms was adopted. These supercell sizes ensure that the results reported in this manuscript are independent of the size of the system. The interatomic interactions in MIL-53­(Al) and COF-5 are described using the QuickFF force fields that were derived and validated previously in ref 53 and ref 26, respectively. To use the Cauchystat implementation in LAMMPS, these force fields were converted to LAMMPS format, using a 15 Å cutoff radius for the long-range interactions and tail corrections. The Coulomb interactions were calculated using a particle–particle particle–mesh (PPPM) solver with an accuracy of 10^–7^.

The MD simulations reported in this work were performed in the (*N*, **σ**, *T*) ensemble and employed the velocity-Verlet time integration of the equations of motion, with a time step of 0.5 fs. Each simulation was performed 10-fold with different random seeds when drawing the initial velocities at 300 K. The temperature during these simulations was controlled at 300 K using a Nosé–Hoover thermostat with a relaxation time of 0.1 ps.^29,54,55^ The Cauchy stress was controlled using the Cauchystat with a relaxation time of 1 ps.^46,47^ All stresses were input in atmospheres in LAMMPS and are shown as such in the figures; to allow comparison with literature, they have been converted to MPa in the text. The parameter *nreset*, which controls after how many steps the reference cell in Figure 2 is reset, is set to 10. However, varying this parameter between its default value of 0 and 1000 gives very similar results, as discussed in Section S2 of the Supporting Information. The control parameter α and the components of the Cauchy stress are varied per case study as mentioned throughout the text.

The results reported herein are derived from MD production simulations of 300 or 200 ps for MIL-53­(Al) and COF-5, respectively. These durations are sufficient to observe whether a given Cauchy stress induces a phase transition and hence to derive the transition stress. For MIL-53­(Al), this production simulation is preceded by an equilibration of 150 ps, during which an isotropic pressure of 0 MPa is applied through the Cauchystat with gradually decreasing values of α: α = 0.1 for the first 18.75 ps, α = 0.01 for the next 18.75 ps, α = 0.001 for the next 37.5 ps, and α = 0.0001 for the last 75 ps of the equilibration, systematically getting closer to the true (*N*, **σ**, *T*) ensemble. Without such equilibration procedure, large stress fluctuations in the initial stages of the MD simulation would induce phase transitions in MIL-53­(Al) even at 0 MPa. In most cases, the proposed four-stage equilibration prevents these transitions from occurring during equilibration; in the infrequent event that a phase transition was still observed during equilibration, such as in Figure S5, the simulation was discarded and restarted with a different random seed. For COF-5, a preceding equilibration was not necessary. Given the large fluctuations in the instantaneous stress—which are also present using hydrostatic barostats^9^—the stress components during an MD simulation reported in this manuscript are first outputted every 5 fs and then averaged over a rolling window with a window size of 1000 frames.

## 3 Results and Discussion

To streamline the discussion, we first discuss normal-stress-induced phase transitions in MIL-53­(Al) in Section 3.1, focusing on the dependence of the critical stress on the direction of the applied normal stress. Subsequently, Section 3.2 examines at which threshold shear stresses in COF-5 lead to layer shearing. Finally, the role of the control parameter α in both transitions is investigated in Section 3.3.

### 3.1 Normal-Stress-Induced Phase Transitions in MIL-53­(Al)

As a starting point, we investigate the MIL-53­(Al) lp-to-cp transition by applying a compressive normal stress in the *z* direction, i.e., only σ_*zz*_ differs from zero and is positive in the production phase. This corresponds with the solid purple lines in Figure 1a. We perform a sweep over the σ_*zz*_ values, repeating each simulation 10-fold with different initial seeds to account for the stochasticity in potential phase transitions.^9^ At this stage, α is maintained at a fixed value of 0.01 throughout the production run.

As discussed in the Computational details, each 300 ps production run is preceded by a four-stage 150 ps equilibration run with a target hydrostatic pressure of 0 MPa. Upon entering each subsequent equilibration stage, indicated by a different shade of gray, the control parameter α is divided by ten, starting from α = 0.1 and ending at α = 0.0001. The necessity for such a trapped equilibration is apparent from Figure 3. The large α values in the initial equilibration stages enable, through eq 2, large updates of the second Piola-Kirchhoff stress and a rapid equilibration. As a result, any deviation between the instantaneous Cauchy stress and the required Cauchy stress observed in the first 37.5 ps of Figure 3d can be quickly corrected by updating the second Piola-Kirchhoff stress, ensuring that the cell vectors in Figure 3a,b evolve around their equilibrium values. In contrast, when entering the third and fourth equilibration stages at 37.5 and 75 ps, the lower α values result in larger deviations between the true stress components and the target stress before they are corrected, as shown in Figure 3d. These deviations furthermore persist over a longer simulation time. As a result, the cell lengths in Figure 3a,b also deviate instantaneously from their equilibrium values, e.g., at around 75 ps. If one would forego this trapped equilibration procedure and immediately start with small α values, these deviations in the true stress would become too large and incorrectly induce phase transformations even at this equilibration stress of 0 MPa. While appropriate equilibration is crucial for any MD simulation, it is essential to note that the range of α values required here goes beyond the default values suggested by LAMMPS.

**Figure 3:**
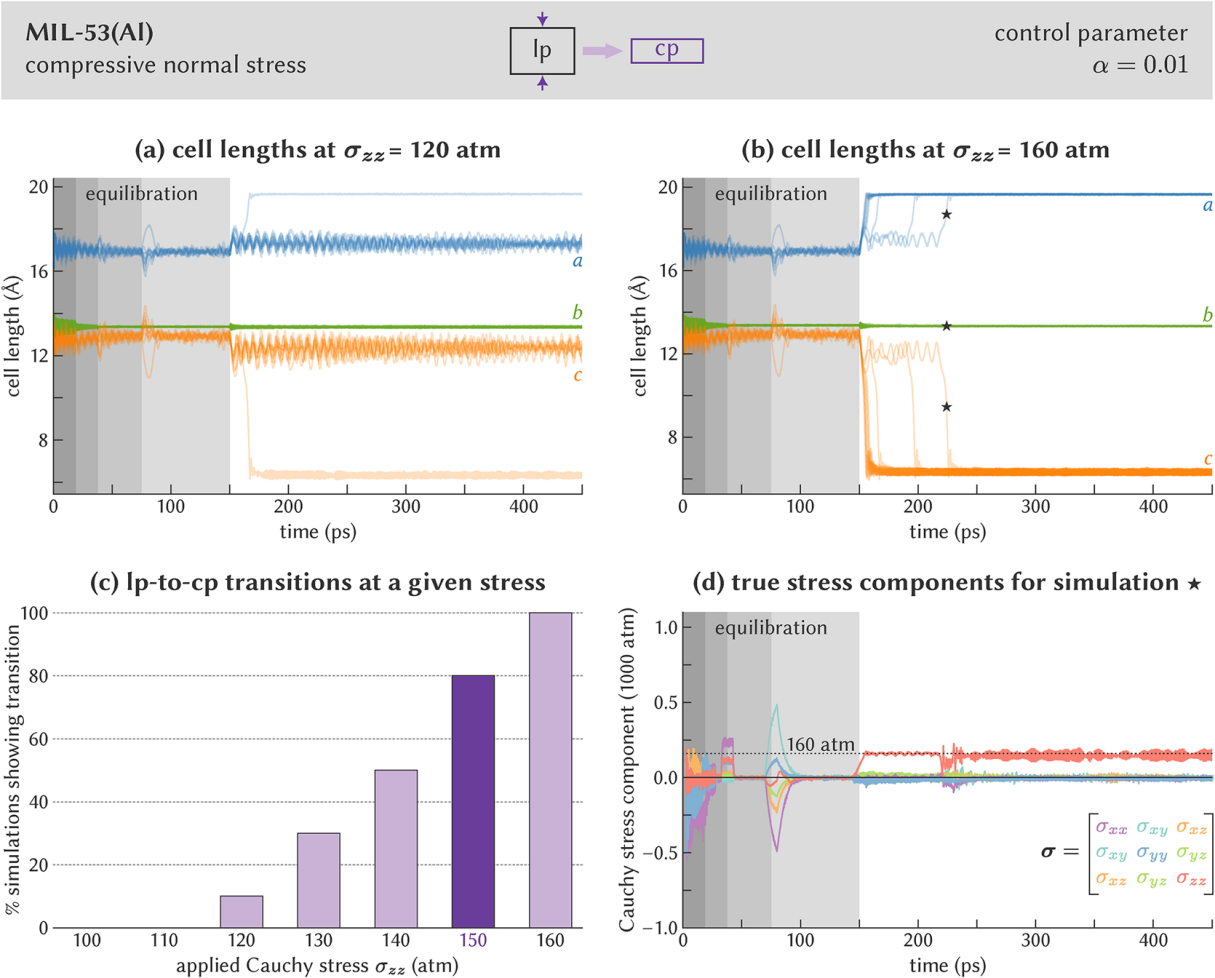
Determination of the σ_*zz*_ critical stress to induce the lp-to-cp phase transition in MIL-53­(Al). (a) Cell lengths for ten independent simulations at σ_*zz*_ = 120 atm, showing that only one simulation undergoes a phase transition. (b) Cell lengths for ten independent simulations at σ_*zz*_ = 160 atm, showing that all simulations undergo a phase transition. (c) Fraction of the simulations that undergo the transition as a function of the applied Cauchy stress. (d) Cauchy stress tensor components during the MD simulation at σ_*zz*_ = 160 atm for which the cell vectors are indicated in (b) with the “★” symbol. In (a), (b), and (d), the first 150 ps correspond with equilibration at a hydrostatic pressure of 0 atm; the nonzero stress is applied from 150 ps onward. In (d), the stress components are averaged over a rolling window with size 5 ps.

Turning our focus to the MIL-53­(Al) behavior when the normal stress is applied after 150 ps, Figure 3a,b contrast the evolution of the cell lengths for the ten independent simulations at σ_*zz*_ ≈ 12 and 16 MPa, respectively. At σ_*zz*_ ≈ 12 MPa, nine out of ten simulations show only a small contraction in the *c* cell length and a small expansion of the *a* cell length, remaining in the slightly contracted lp phase for the whole 300 ps. For the tenth simulation in Figure 3a, an lp-to-cp transition is observed. This stochastically induced phase transition near the transition stress is expected, as these stochastic transitions also occur under hydrostatic pressures due to instantaneous stress fluctuations.^9^ Importantly, these stochastic events occur only infrequently, in contrast with the situation at σ_*zz*_ ≈ 16 MPa, for which all ten simulations undergo an lp-to-cp transition. Repeating this procedure for different normal stresses yields the transition statistics visualized in Figure 3c. For stresses below 12 MPa, all simulations remain in the lp phase, while all simulations undergo a transition to the cp phase at a stress of 16 MPa or larger. In the range of 12 to 15 MPa, stochastic transitions occur.

From Figure 3c, one can define the lp-to-cp critical stress as the lowest stress at which more than half of the simulations undergo an lp-to-cp transition. This results in a critical σ_*zz*_ stress of approximately 15 MPa, which is substantially below the hydrostatic transition pressure of 25–30 MPa (see Section S1 of the Supporting Information). Importantly, we do not expect the predicted transition stress to change by more than a few MPa when considering longer simulations since we performed ten independent simulations at each stress value. The observation that the lp-to-cp critical stress is substantially lower than the lp-to-cp transition pressure can be rationalized by the fact that, in contrast to the aforementioned normal stress, a hydrostatic pressure tries to compress not only the *c* but also the *b* and *a* cell lengths, which should remain constant and expand, respectively, when undergoing the lp-to-cp transition. As a result, applying anisotropic stresses to MIL-53­(Al) would lead to a different transition behavior compared to merely applying hydrostatic pressures.

Finally, Figure 3d visualizes the evolution of the instantaneous Cauchy stress during the simulation at around 16 MPa that is indicated by the star in Figure 3b. It demonstrates that, when applying a nonzero stress state at 150 ps, the six independent stress components evolve quickly toward, and then fluctuate around, their target values. Around 220 ps, Figure 3b indicates that the lp-to-cp transition occurs, with a substantial change in the cell parameters and hence the deformation gradient **F**. However, the control parameter α is sufficiently large to limit the impact on the instantaneous Cauchy stress, which shows only minor fluctuations around the 220 ps timestamp in Figure 3d before returning to its equilibrium value from 250 ps onward.

Inspired by this decrease in stress necessary to induce an lp-to-cp transition when uniaxially compressing the material compared to a hydrostatic pressure, we explore whether the same observation holds when subjecting MIL-53­(Al) to a tensile normal stress. As indicated in Figure 1a, a tensile σ_*xx*_ stress, shown with dotted purple arrows, should induce the same lp-to-cp transition as the compressive σ_*zz*_ stress discussed before, shown with solid purple arrows, but this time primarily elongating the *a* cell length instead of compressing the *c* cell length.

Figure 4a,b visualize the evolution of the cell lengths during a simulation at σ_*xx*_ ≈ −20 MPa and σ_*xx*_ ≈ −40 MPa. At a stress of approximately −20 MPa, all simulations remain in the lp phase, whereas applying a larger tensile stress of approximately −40 MPa is sufficient to induce an lp-to-cp phase transition in nine out of ten repeated simulations. As for the compressive stress, Figure 4d evidences that a similar equilibration procedure is needed to keep the stress fluctuations under control.

**Figure 4:**
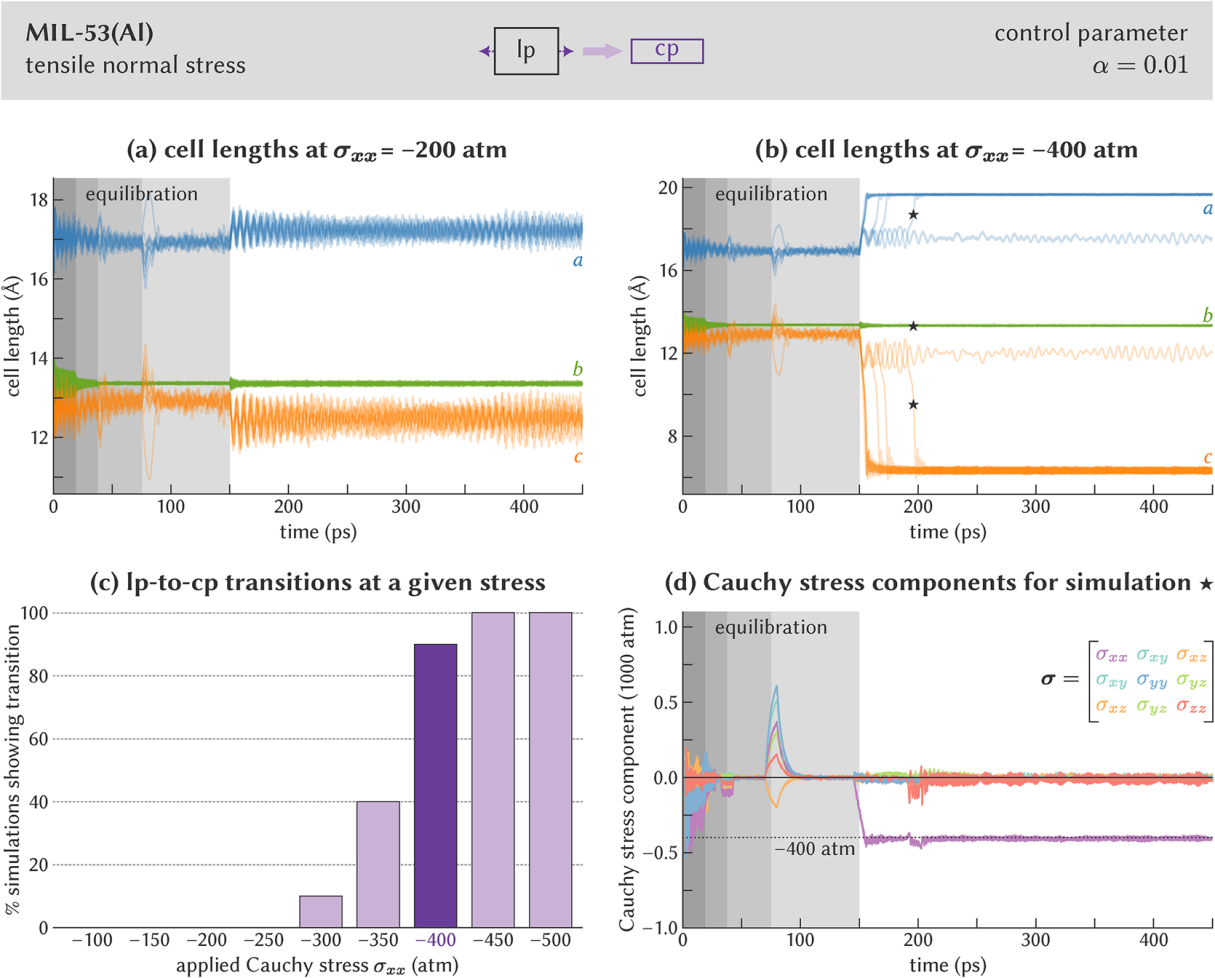
Determination of the σ_*xx*_ critical stress to induce the lp-to-cp phase transition in MIL-53­(Al). (a) Cell lengths for ten independent simulations at σ_*xx*_ = −200 atm, showing that none of the simulations undergo a phase transition. (b) Cell lengths for ten independent simulations at σ_*xx*_ = −400 atm, showing that nine out of ten simulations undergo a phase transition. (c) Fraction of the simulations that undergo the transition as a function of the applied Cauchy stress. (d) Cauchy stress tensor components during the MD simulation at σ_*xx*_ = −400 atm for which the cell vectors are indicated in (b) with the “★” symbol. In (a), (b), and (d), the first 150 ps correspond with equilibration at a hydrostatic pressure of 0 atm; the nonzero stress is applied from 150 ps onward. In (d), the stress components are averaged over a rolling window with size 5 ps.

A full stress sweep, with transition statistics depicted in Figure 4c, demonstrates that the critical σ_*xx*_ stress lies around −40 MPa, which is larger in magnitude than both the critical hydrostatic pressure and the critical σ_*zz*_ stress found before. This directional dependence of the critical stress can be explained by the smaller unit cell area on which the σ_*xx*_ stress acts, which is mainly defined by the *b* and *c* cell lengths, compared to the σ_*zz*_ stress, for which the area is primarily defined by the *a* and *b* cell lengths. As a result, a comprehensive anisotropic investigation, such as the one carried out here, is necessary to appreciate the extent of anisotropy present in these materials, especially when they are adopted in applications such as nanosensing or -damping, where a hydrostatic loading cannot be assumed *a priori*. In Section S3 of the Supporting Information, we also explore the MIL-53­(Al) response to a compressive σ_*xx*_ or a tensile σ_*zz*_ stress, corresponding to the brown arrows in Figure 1a, further highlighting this importance.

### 3.2 Shear-Stress-Induced Layer Instability in COF-5

To investigate the ability of the Cauchystat also to control shear stresses, we consider three different shear stresses in the layered COF-5. In all cases, the stress is applied on a plane normal to the *z* axis, with a force that points either (i) parallel to the *y* axis (a σ_*yz*_ stress, shown in Figure 1b), (ii) parallel to the *x* axis (a σ_*xy*_ stress), or (iii) parallel to the direction in the *xy* plane that makes an angle of 30° with the *x* axis (denoted the σ_30°_ stress). Due to the hexagonal symmetry of COF-5, this latter direction is equivalent to the first one and should yield the same transition stress.

The evolution of the three cell angles in Figure 5a at a stress σ_*yz*_ ≈ 15 MPa shows the typical behavior at low stresses. The out-of-plane β and in-plane γ angles oscillate around their initial values of 90° and 120°, respectively. In contrast, the out-of-plane α angle between the ***b*** and ***c*** vectors quickly increases from the initial AA-stacking value at 90° to ca. 120°, leading to the inclined configuration of Figure 1b. This shearing transition is expected, as both experiments and simulations indicate that the inclined stacking configuration is approximately 15 kJ·mol^–1^ more favorable at 300 K compared to the AA-configuration,^17^ primarily due to Pauli repulsion.^16^

**Figure 5:**
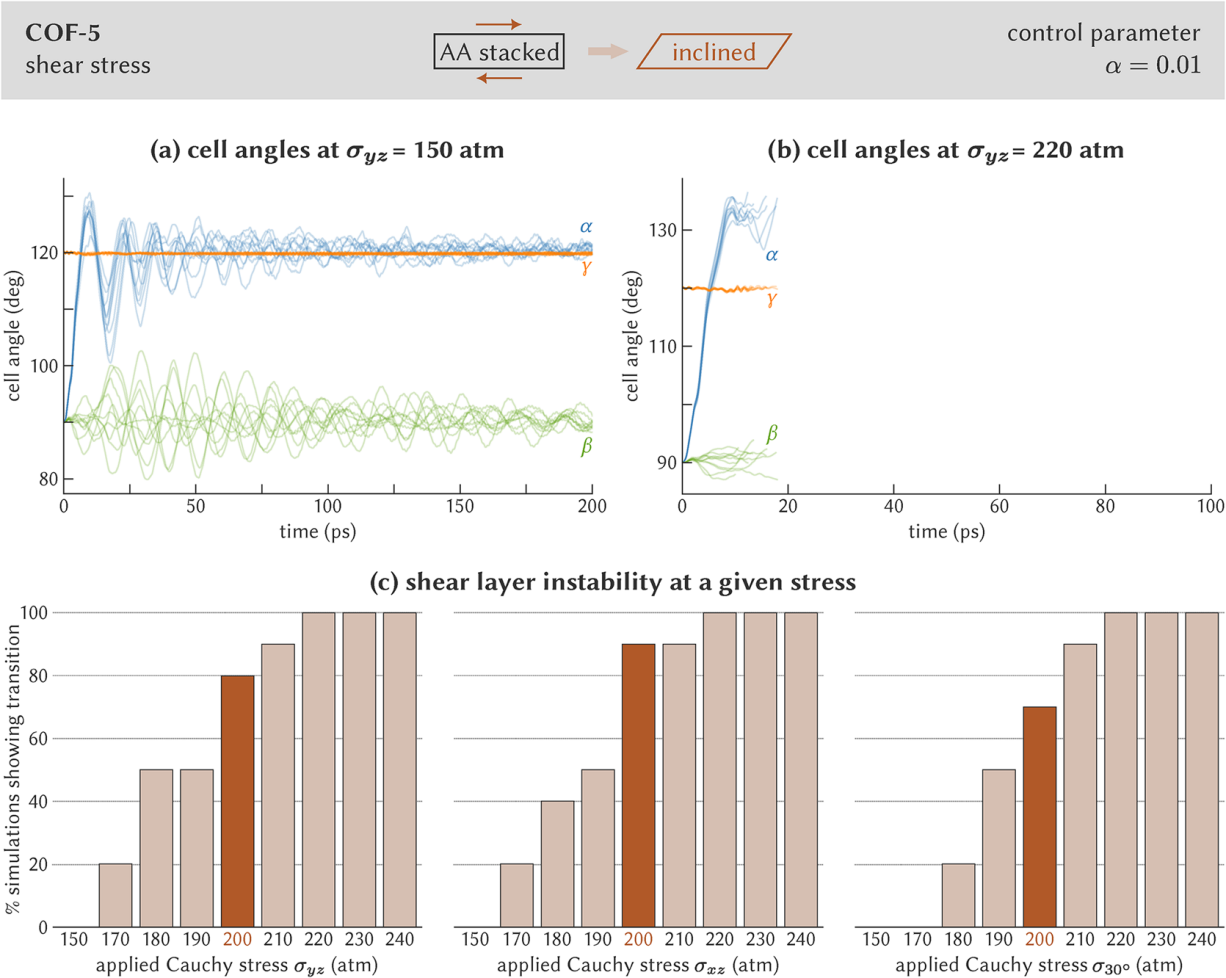
Determination of the critical shear stress to induce a layer shearing instability in COF-5. (a) Cell angles for ten independent simulations at σ_*yz*_ = 150 atm, showing that none of the simulations undergo a layer shearing instability. (b) Cell angles for ten independent simulations at σ_*yz*_ = 220 atm, showing that all simulations undergo a layer shearing instability. (c) Fraction of the simulations that undergo the transition as a function of the applied Cauchy stress. Three stresses are considered, all acting on a plane perpendicular to the *z* direction: σ_*yz*_ acts parallel with the *y* axis, σ_*xz*_ acts parallel with the *x* axis, and σ_30°_ acts parallel with the direction in the *xy* plane making an angle of 30° with the *x* axis.

According to ref 17, trying to shear adjacent layers beyond the inclined stacking observed above would require the material to overcome a free energy barrier of about 120 kJ·mol^–1^ at 300 K. In that case, the layers become disconnected, leading to an uncontrolled shearing instability through delamination. Since such substantial energy can, in principle, be supplied via a shear deformation, we further investigated COF-5′s response to higher shear stresses. Taking the σ_*yz*_ ≈ 22 MPa case shown in Figure 5b as an example, one systematically observes that a sufficiently high shear stress indeed steers the cell angle α beyond 120°. In our simulations, once the cell angle exceeds approximately 130°, the layers fully shear with respect to one another. After this point, the simulation ends, as the layers would continue to shear indefinitely under this stress value otherwise.

Similar to MIL-53­(Al), we performed ten independent runs at each shear stress to collect statistics. Figure 5c illustrates that the aforementioned shear layer instability occurs with increasing frequency from σ_*yz*_ ≈ 17 MPa onward, with a critical stress of about 20 MPa. A similar critical stress is observed in Figure 5c when a σ_*xz*_ or σ_30°_ shear stress is applied. However, it is the β angle, rather than the α angle, that exceeds 130° and leads to delamination in these cases. While the σ_*yz*_ and σ_30°_ stresses are related due to COF-5′s symmetry, obtaining the same critical σ_*xz*_ stress is not dictated by symmetry. This indicates that the shearing motion in COF-5 occurs largely independent of the direction of the shearing force, as long as it occurs parallel to the layers. A larger anisotropy could be observed for less-symmetric 2D COFs.

### 3.3 Influence of the Control Parameter **α**

Until now, we performed all production simulations with a control parameter α of 0.01, in line with the 0.01–0.001 range suggested by LAMMPS. However, smaller α values are preferred, since the correct thermodynamic stress fluctuations are only ensured in the limit α → 0.^46^ In contrast, the equilibration procedure in MIL-53­(Al) clearly demonstrates that large values of α are required to prevent significant deviations between the target Cauchy stress and its instantaneous values, thereby avoiding incorrect phase transitions. For this reason, we here varied the control parameter α over several orders of magnitude, focusing on the σ_*zz*_ induced lp-to-cp transition in MIL-53­(Al) and the σ_*yz*_ induced layer shearing instability in COF-5.

Figure 6a,b summarize the results of this α exploration for MIL-53­(Al) and COF-5, respectively. When adopting our usual definition, the predicted critical stress varies barely, both in MIL-53­(Al)—between 15 and 16 MPa—and in COF-5—between 18 and 21 MPa. However, in line with our earlier observations during the equilibration of MIL-53­(Al), the magnitude of α does impact the sharpness with which the transition stress can be defined. For instance, compare α = 0.1 with α = 0.0001 in Figure 6a. In the former case, all simulations at a given stress magnitude either remain in the lp phase or undergo an lp-to-cp transition, except for a small stress range between approximately 13 and 15 MPa. Between these stress values, stochastic fluctuations in the Cauchy stress may induce phase transitions in one simulation, while leaving another simulation at the same stress in the lp phase. This small stress range, in which transitions may or may not occur, increases as α is decreased. For α = 0.0001, we observed stochastic transitions even at pressures as low as 0 MPa, up to approximately 17 MPa. This observation directly follows from eq 2, since stochastic fluctuations in the Cauchy stress are more easily corrected at large α values by adapting the second Piola-Kirchhoff stress. For COF-5, shown in Figure 6b, the effect of α on the range of stresses for which stochastic transitions are observed is less pronounced, although COF-5 simulations at α = 0.0001 became unstable. As a result, choosing an appropriate value for the control parameter is important both during the equilibration run—to prevent premature phase transitions—and the actual production run—to accurately pinpoint the transition stress.

**Figure 6:**
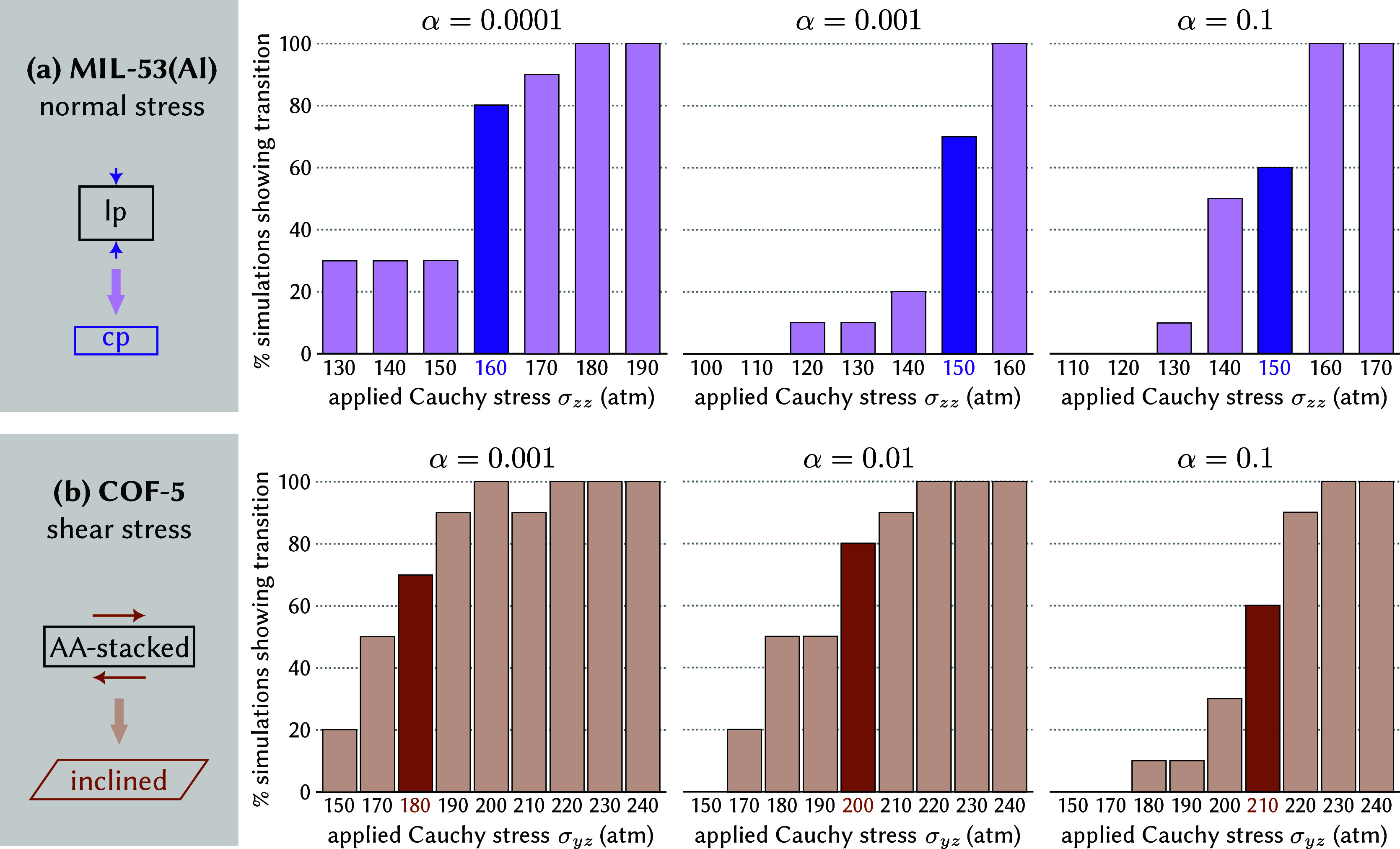
Influence of the strength of the control parameter α on the stress threshold (a) for an lp-to-cp transition in MIL-53­(Al) and (b) for a layer shearing instability in COF-5. The lowest stress for which more than five out of ten independent simulations undergo a transition is highlighted.

## 4 Conclusion

Having presented herein the first application of the Cauchystat to steer stress-induced phase transitions in soft porous crystals, we take this opportunity to formulate the following best practices for investigating soft anisotropic materials, in addition to the original suggestions by Miller et al.^46^

First, our simulations on MIL-53­(Al) and COF-5 confirmed that, also for these highly stimuli-sensitive materials, the Cauchystat is able to predict stress-induced phase transitions. The method succeeds in inducing both the MIL-53­(Al) lp-to-cp transition through normal stresses and the COF-5 layer instability through shear stresses. While the first transition was studied extensively before using hydrostatic pressures, the latter was, until now, characterized only through free energy surfaces, which left open the question of the critical stress needed to induce the instability. Both case studies exemplify the need to characterize the response of these materials to an anisotropic mechanical stimulus, rather than relying solely on hydrostatic pressures.

Second, applying anisotropic stresses invokes material responses that differ from those induced by a simple hydrostatic pressure. For instance, for MIL-53­(Al), we found that applying a normal stress in different directions substantially altered the critical stress necessary to induce the lp-to-cp transition, which could be rationalized based on the different areas on which the stress is applied. In contrast, due to its higher symmetry, the critical stress to induce a shear layer instability in COF-5 is largely direction-independent. Importantly, for MIL-53­(Al), the predicted critical normal stresses differ from the critical hydrostatic pressure, which can be explained by the anisotropy during the lp-to-cp transition. Hence, for applications that rely on the stimuli-responsiveness of these nanoporous materials, it is crucial to investigate their response under all independent stress stimuli rather than considering hydrostatic pressures alone.

Third, the control parameter α plays a crucial role in taming deviations between the target Cauchy stress and fluctuations in the instantaneous Cauchy stress. A higher value of α, and hence a stronger Cauchy control, is especially needed to remove any initial stresses during equilibration, as observed for MIL-53­(Al), as well as to pinpoint the transition stress more accurately. However, as mentioned before, the correct stress fluctuations are only retrieved in the limit α → 0.^46^ The parameter *nreset* has a much smaller impact on this.

In conclusion, while this manuscript illustrates the importance of anisotropic stress control in soft materials and the validity of the Cauchystat, it also points toward potential improvements. Since the Cauchystat acts as a control algorithm that continuously adapts the applied second Piola-Kirchhoff stress through eq 2, it disturbs the equilibrium stress distribution dictated by thermodynamics, leading to complex equilibration and production runs with a varying control parameter α to reduce these disturbances. This contrasts with the original Parrinello–Rahman barostat, which is based on the extended Hamiltonian approach and directly yields a set of equations of motion with a conserved quantity. Yet, this barostat does not control the Cauchy stress directly and therefore does not sample the (*N*, **σ**, *T*) ensemble. As a result, it remains an open question whether a similar extended Hamiltonian approach can be followed to directly control the Cauchy stress, thereby combining the advantages of both methods.

## Supplementary Material



## Data Availability

The LAMMPS force field files and representative input scripts are available at https://github.com/SvenRogge/supporting-info.

## Notes

Selection of the supercell size, choice of reset frequency for the reference cell, discussion of the transitions in MIL-53 leading to the overstretched large pore phase (PDF)
