# Supplementary material for: Challenges and Best Practices in Modeling Anisotropic Stresses in Soft Polymorphic Materials
Source: ACS Phys Chem Au. 2026 Jan 8;6(2):356–66. doi: 10.1021/acsphyschemau.5c00141 (PMC13022792; doi:10.1021/acsphyschemau.5c00141)
Supplement: Supplementary file 1 [file pg5c00141_si_001.pdf]

Supporting Information for

Challenges and Best Practices in Modeling Anisotropic Stresses in Soft Polymorphic Materials

Jelto Neirynck, Sander Geerinckx, and Sven M. J. Rogge*

Center for Molecular Modeling (CMM), Ghent University, Technologiepark-Zwijnaarde 46, 9052 Zwijnaarde, Belgium

E-mail: Sven.Rogge@UGent.be

S1 Choice of appropriate supercell size **S-2**

S2 Influence of the reference cell reset frequency **S-4**

S3 Normal-stress-induced transitions between the large pore and overstretched large pore phase in MIL-53(Al) **S-7**

S3.1 Compressive σ_{xx} stress S-7

S3.2 Tensile σ_{zz} stress S-9

S1 Choice of appropriate supercell size

The instantaneous pressure fluctuates strongly during molecular dynamics (MD) simulations, which lies at the origin of premature phase transitions.[1] For MIL-53(Al), we demonstrated that even when the applied pressure is below the transition threshold, phase transitions can be induced due to large fluctuations in the instantaneous pressure that exceed the transition pressure threshold for a sufficiently long time.[1] While one can avoid these premature phase transitions altogether by adopting the $(N, V, \boldsymbol{\sigma}_a = \mathbf{0}, T)$ ensemble, with $\boldsymbol{\sigma}_a$ the deviatoric stress,[1] we cannot follow that approach in this work since we explicitly aim to model transition stresses in the $(N, \boldsymbol{\sigma}, T)$ ensemble. However, an alternative approach lies in considering large enough simulation cells. Because the relative fluctuations in instantaneous pressure scale as $N^{-1/2}$, with N the number of atoms in the simulation cell,[2] premature phase transitions will be suppressed for larger cells.

To investigate the simulation cell size required to prevent premature phase transitions, we performed simulations on the traditional 1×2×1 simulation cell of MIL-53(Al), as well as the 2×4×2, 3×6×3, and 4×8×4 supercells. For each pressure and supercell, we performed ten independent simulations with different random seeds. 450 ps production runs were performed with the same thermostat settings as in the main text, but without *prior* equilibration and using a Martyna–Tobias–Tuckerman–Klein barostat with a relaxation time of 1 ps to apply a hydrostatic pressure.[3,4]

As shown in Figure S1, the traditional 1×2×1 simulation cell predicts a negative lp-to-cp transition pressure of about –10 MPa, which is substantially different from the value of 27 to 30 MPa found earlier with a similar force field.[1] Only for simulation cells at least as large as the 3×6×3 simulation cell is this correct transition pressure retrieved. Assuming that a similarly sized cell is needed to avoid premature phase transitions due to anisotropic stress fluctuations, this 3×6×3 simulation cell will be used to model MIL-53(Al) in the main paper. Since no reference studies exist for the critical shear stress in COF-5, we will use a larger 4×4×4 simulation cell in that case.

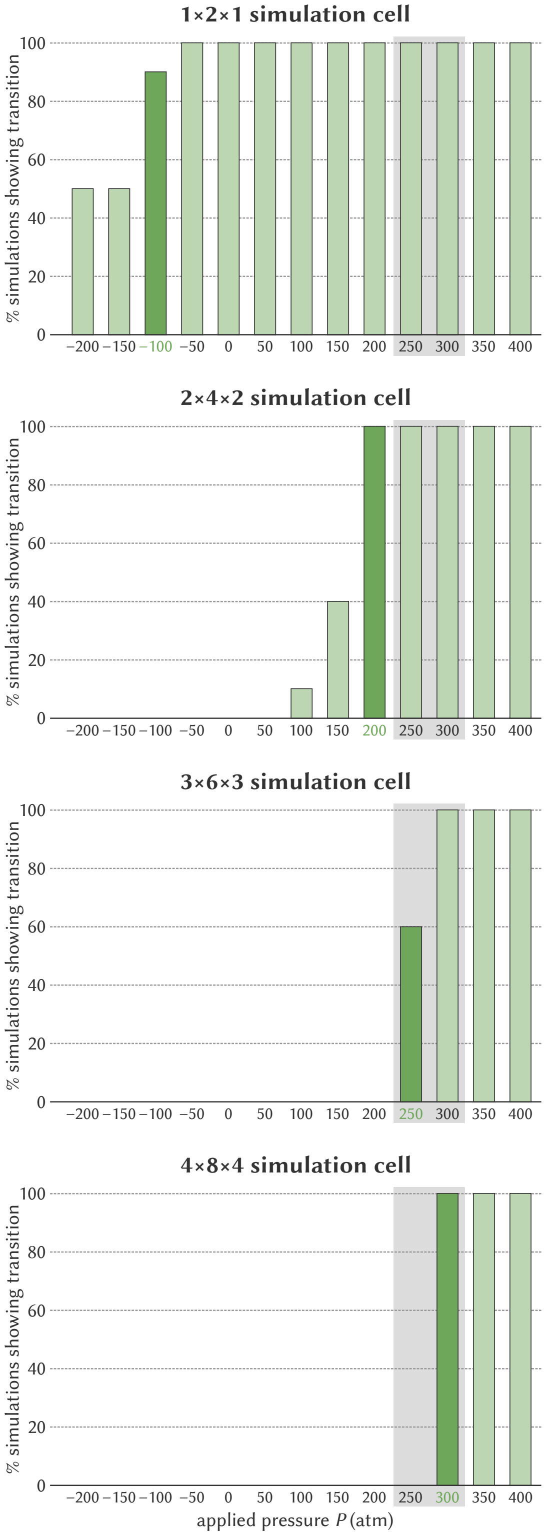

Figure S1: Fraction of the simulations that undergo an lp-to-cp transition as a function of the applied hydrostatic pressure for four different simulation cell sizes. The grey background indicates the transition pressure range found earlier.[1]

S2 Influence of the reference cell reset frequency

The Cauchystat *nreset* parameter determines the number of MD steps after which the reference cell $\mathbf{h}_0$ is redefined. If *nreset* > 0, the reference cell is changed every *nreset* steps and defined to correspond to the instantaneous cell matrix $\mathbf{h}$ at that time instant. If *nreset* $= 0$, which is the default in LAMMPS, the reference cell is not redefined during the simulation (counterintuitively, this corresponds with *nreset* $\rightarrow \infty$). Through Figure 2 of the main text, the reference cell enters the deformation gradient and, *via* Eq. 1 of the main text, defines the relation between the different stress tensors. In principle, the definition of this reference cell is arbitrary. In practice, however, significant deviations between the instantaneous cell matrix and the reference cell matrix will inflate the importance of the first term between brackets in Eq. 2 of the main text, at the expense of the second term. As a result, the Cauchystat control is expected to be less sensitive in steering the instantaneous stress towards the required applied stress when the reference cell is too different from the cell matrices one is encountering during the simulation.

Therefore, we opted to investigate the influence of *nreset* on the different transition stresses discussed in the main text. To this end, we kept *nreset* equal to 10 during the equilibration period, but varied its value during the production run. Figure S2 demonstrates that this parameter has a minor impact on the predicted σ_{zz} transition stress to induce an lp-to-cp transition in MIL-53(Al): all predicted critical stresses vary between 14 and 15 MPa, regardless of whether *nreset* adopts its default value of 0 or whether the reference cell is redefined during the simulation. A similar insensitivity to the *nreset* parameter also holds for the other normal stresses in MIL-53(Al) and the shear stresses in COF-5.

Our decision to set *nreset* to 10, rather than the default of 0, is motivated by Figure S3. This figure contrasts the Cauchy stress components and cell lengths at two MD simulations with $\sigma_{zz} \approx 16$ MPa. On the left in Figure S3, the simulation is performed with *nreset* $= 10$, which was discussed earlier in Figures 3b and 3d of the main text. On the right in Figure S3, the simulation is performed with *nreset* $= 0$ instead. While both simulations predict the same lp-to-cp transition pressure and retrieve similar cell parameters for the lp and cp phases, the instantaneous fluctuations in the Cauchy stress components are larger in the *nreset* $= 0$ case after the lp-to-cp transition at around 175 ps. As discussed above, we hypothesise that this is

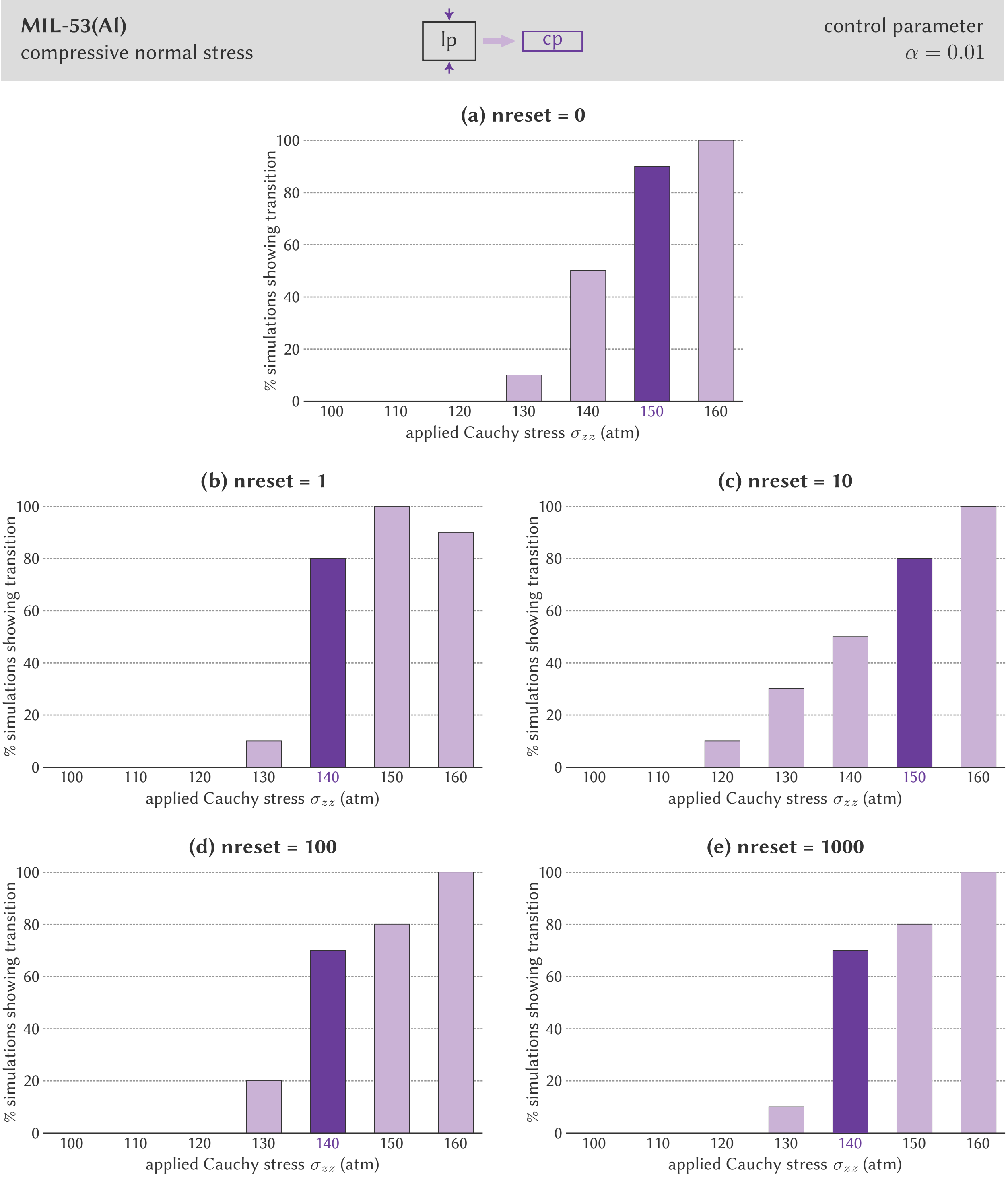

Figure S2: Fraction of the simulations that undergo an lp-to-cp transition as a function of the applied σ_{zz} stress for five different values of the *nreset* parameter during the production run. Note that panel c, with *nreset* $= 10$, is reproduced in Figure 3c of the main text.

the result of the first term in Eq. 2 in the main text dominating over the second term, which controls the stress fluctuations, due to the significant difference between the reference cell and the actual simulation cell after the transition. For *nreset* = 10, this difference disappears when a new reference cell is chosen throughout the simulation. In contrast, for *nreset* = 0, the original reference cell is retained throughout the whole simulation, and the larger fluctuations persist. Therefore, we chose *nreset* = 10 for all simulations in the main text.

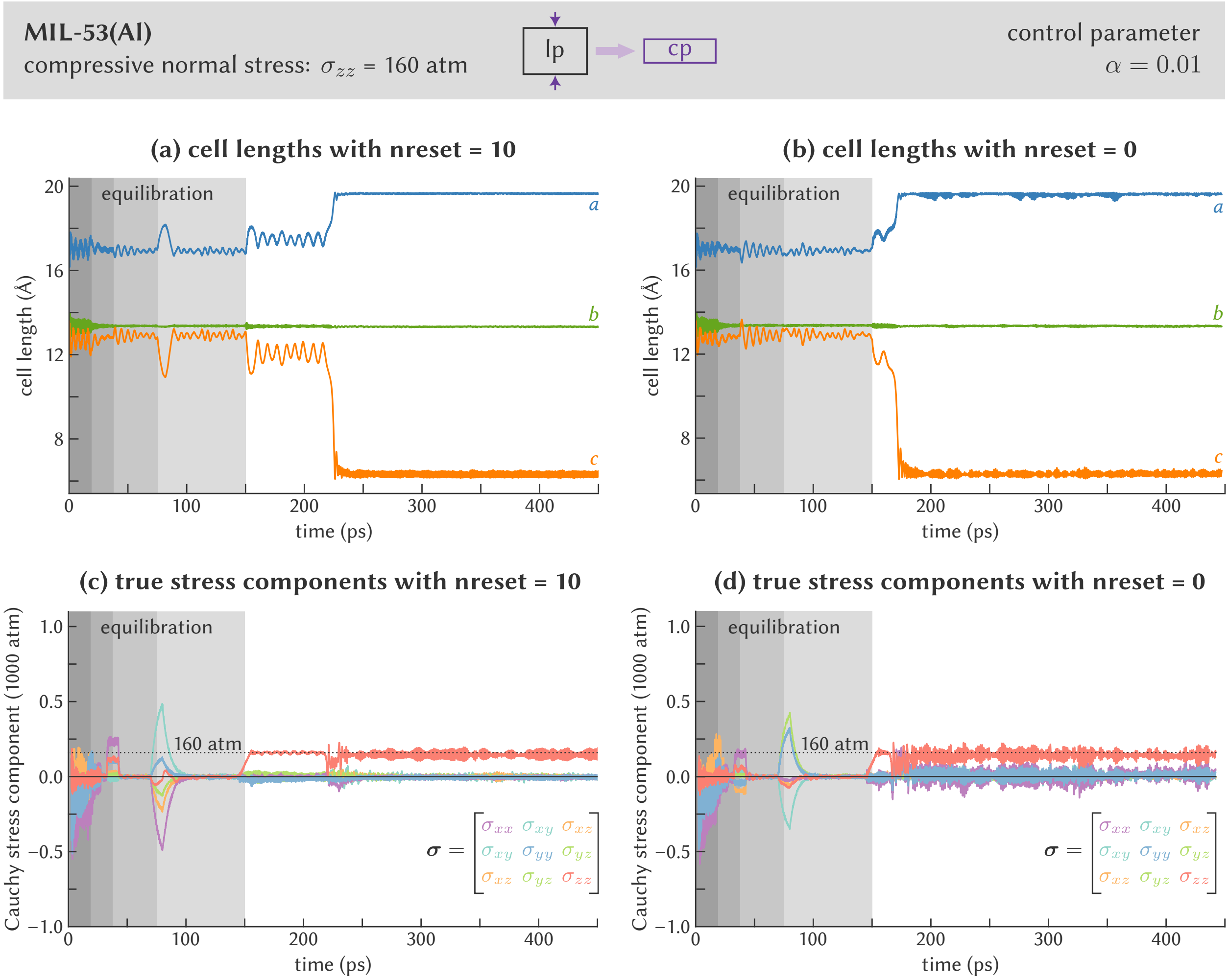

Figure S3: Influence of the choice of the *nreset* parameter on the MIL-53(Al) cell lengths and stress fluctuations, illustrated for a simulation at σ_{zz} = 160 atm. Panels a and c show the cell lengths and instantaneous true stress for the simulation indicated with the $\star$ symbol in Figure 3b of the main text, which was calculated with *nreset* = 10. Panels b and d show the cell lengths and instantaneous true stress for the simulation in which the parameter *nreset* was set to zero.

S3 Normal-stress-induced transitions between the large pore and overstretched large pore phase in MIL-53(Al)

In the main text, we discussed the lp-to-cp transitions observed when subjecting the MIL-53(Al) lp phase to either a sufficiently large compressive σ_{zz} stress or a sufficiently large tensile σ_{xx} stress, indicated by the purple arrows in Figure 1b of the main text. In this Supplementary Section, we investigate what happens when switching the sign of the stress, *i.e.*, how the lp phase evolves when applying a tensile σ_{zz} or a compressive σ_{xx} stress. As anticipated with the brown arrows in Figure 1a of the main text, we expect to encounter an overstretched lp phase in this case.

S3.1 Compressive σ_{xx} stress

Figure S4 summarises the evolution of the MIL-53(Al) cell lengths when applying a compressive σ_{xx} stress with varying magnitude. Independent of the magnitude of this compressive stress, MIL-53(Al) responds by compressing the a cell length and expanding the c cell length, while the b cell length remains approximately unchanged compared to the lp phase. When increasing the magnitude of the compressive σ_{xx} stress, Figure S4 reveals that the a and c cell lengths further compress and expand, respectively, without leading to a well-defined phase transition. This behaviour contrasts with the cp phase induced under a tensile σ_{xx} stress, which is characterised by a narrow range of cell parameters, but is expected given the absence of a separate free energy minimum corresponding to this overstretched large pore (olp) structure.[1] The contrast between the cp and olp phase behaviour arises from the presence of the μ_2-OH groups in the MIL-53(Al) aluminium hydroxide backbone. In the cp phase, the μ_2-OH proton is located in the larger angle of the lozenge pore shape. In contrast, in the olp phase, it is located in the smaller angle, which leads to repulsion between this proton and the neighbouring linkers in the structure, destabilising a potential olp phase. For this reason, no well-defined transition pressure can be determined. We note, however, that at a compressive σ_{xx} stress of about 130 MPa, the a and c cell lengths have a similar magnitude.

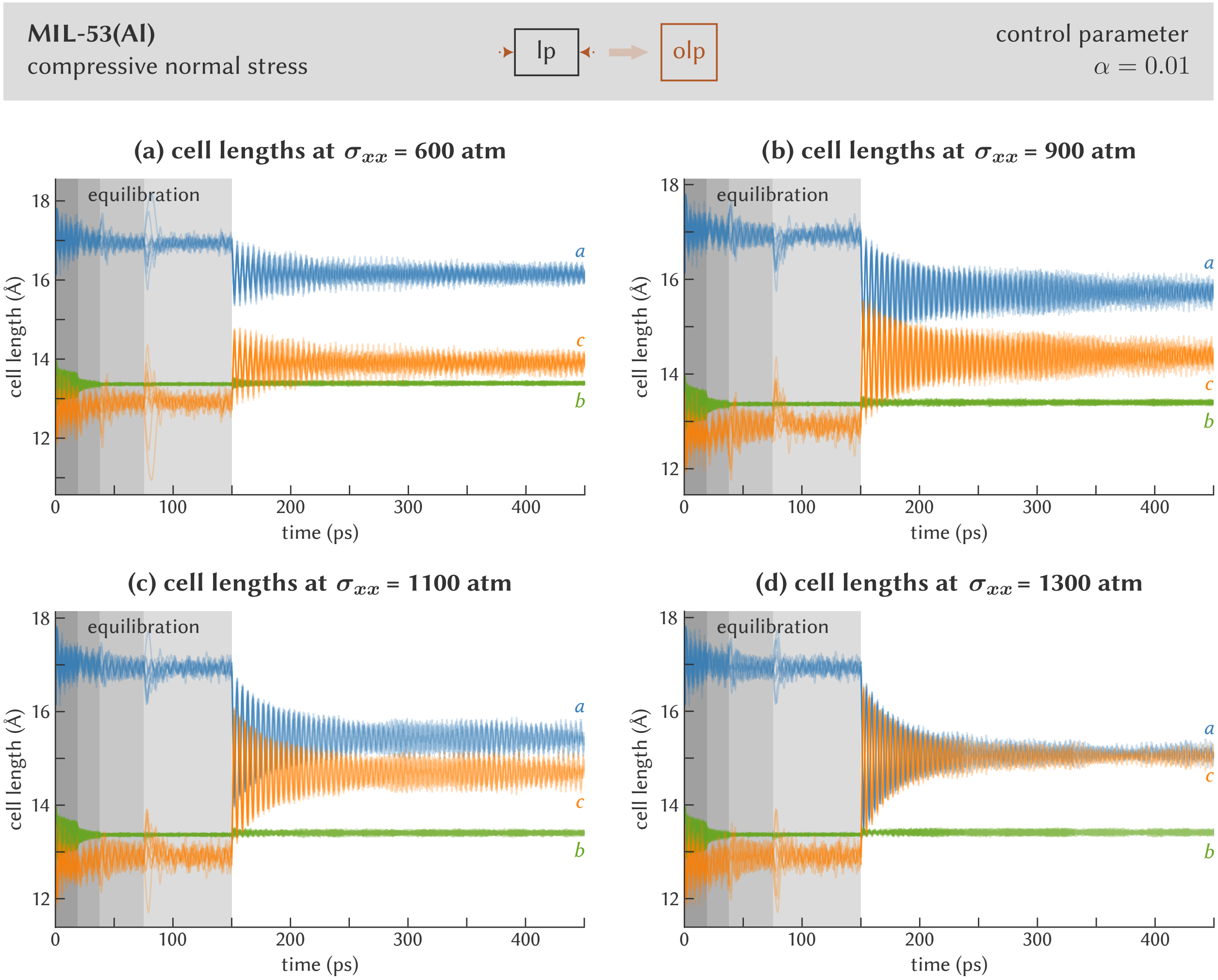

Figure S4: Evolution of the cell lengths for ten independent simulations at σ_{xx} = **(a)** 600 atm, **(b)** 900 atm, **(c)** 1100 atm, and **(d)** 1300 atm. As in the main text, the first 150 ps correspond with equilibration at a hydrostatic pressure of 0 atm; the non-zero stress is applied from 150 ps onwards.

S3.2 Tensile σ_{zz} stress

The olp structures found under a compressive σ_{xx} stress can also be obtained under a tensile σ_{zz} stress, as shown in Figure S5. A continuous evolution of the cell lengths as a function of the stress is observed, similar to Figure S4. At a σ_{zz} stress of about –130 MPa, the a and c cell lengths coincide, which is the same magnitude as the compressive σ_{xx} stress needed to obtain this structure. This contrasts with the different magnitude of the compressive σ_{zz} *versus* the tensile σ_{xx} stress required to induce an lp-to-cp transition. In the main text, we hypothesised that the origin of this difference arises from the difference in area on which these stresses act in the lp

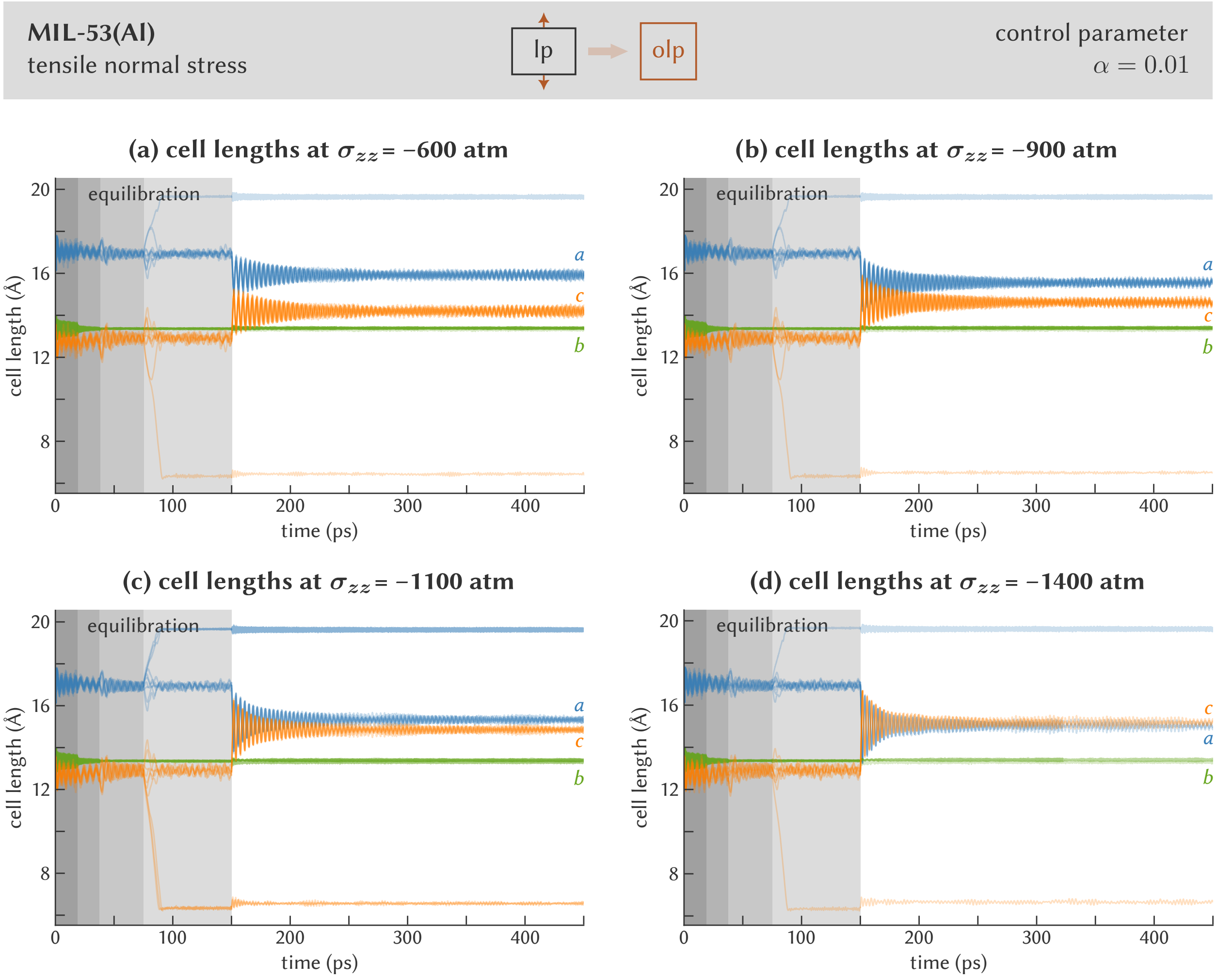

Figure S5: Evolution of the cell lengths for ten independent simulations at σ_{zz} = **(a)** –600 atm, **(b)** –900 atm, **(c)** –1100 atm, and **(d)** –1400 atm. As in the main text, the first 150 ps correspond with equilibration at a hydrostatic pressure of 0 atm; the non-zero stress is applied from 150 ps onwards.

phase. In the olp structures at approximately 130 MPa shown in Figures S4 and S5, however, $a \approx c$, resulting in a similar area on which these stresses act on the olp phase, and hence a similar stress is needed to observe the same behaviour.

Finally, we note in Figure S5 that, despite the extensive equilibration procedure, some lp simulations undergo a transition to the cp phase during equilibration, when the hydrostatic pressure is controlled to be 0 MPa. This further underscores the importance of selecting appropriate values for the Cauchystat control parameter α.

References

(1) Rogge, S. M. J.; Vanduyfhuys, L.; Ghysels, A.; Waroquier, M.; Verstraelen, T.; Maurin, G.; Van Speybroeck, V. A Comparison of Barostats for the Mechanical Characterization of Metal–Organic Frameworks. *J. Chem. Theory Comput.* **2015**, *11*, 5583–5597.

(2) Landau, L. D.; Lifschitz, E. M. *Volume 5: Statistical Physics*, 3rd ed.; Elsevier Butterworth-Heinemann: Oxford, 1980.

(3) Martyna, G. J.; Tobias, D. J.; Klein, M. L. Constant Pressure Molecular Dynamics Algorithms. *J. Chem. Phys.* **1994**, *101*, 4177–4189.

(4) Martyna, G. J.; Tuckerman, M. E.; Tobias, D. J.; Klein, M. L. Explicit Reversible Integrators for Extended Systems Dynamics. *Mol. Phys.* **1996**, *87*, 1117–1157.
